# High Prevalence of Dyslipidemia in Children and Adolescents: Opportunity for Prevention

**DOI:** 10.36660/abc.20190761

**Published:** 2020-01

**Authors:** Leonardo Mangili

**Affiliations:** Parana Medical Research Center (PMRC), Maringá, PR - Brazil

**Keywords:** Cardiovascular Diseases, Dyslipidemias, Hypercholesterolemi, Child, Young Adult, Unified Health System, Adolescent, Laboratoty Test

Cardiovacular diseases are a major cause of morbidity and mortality. According with the World Health Organization (WHO), worldwide, one third of ischemic heart diseases are attributable to high cholesterol, which causes 2.6 million deaths per year.^1^ Atherosclerosis begins in childhood and evolves in an insidious process which can last decades from the first artery injuries to the clinical outcomes (death, myocardial infarction or strokes). This process is speeded up by risk factors such as cholesterol, smoking, obesity and hypertension.^2^

The multicenter study Pathological Determinants of Atherosclerosis in Youth (PDAY) revealed the presence of atherosclerosis injuries in all aortas and in 50% of right coronary arteries in 1.532 necropsies of individuals aged from 15 to 19 years.^3^ When the role of risk factors was assessed in individuals between 15 and 34 years of age, it was found that the aortic injuries were positively correlated with LDL and VLDL levels, glucose intolerance, smoking, hypertension, obesity, but negatively associated with HDL levels.^4^ Similar findings were described by the Bogalusa Heart Study, which associated the presence of fatty streaks in the aorta with higher total and LDL cholesterol levels, in addition to an inverse association with HDL-C.^5^ There was also greater severity of atherosclerosis injuries in the presence of multiple concomitant risk factors (body mass index, blood pressure, cholesterol and triglyceride concentration).^6^

In agreement with the evidence that increased cholesterol levels promote atherosclerosis, Mendelian randomized studies demonstrate that exposure to genetically lower cholesterol levels since childhood is associated with a reduction in the risk for coronary artery disease (CAD). It was estimated that for each 1 mmol/l (38.7 mg/dl) reduction in LDL, there is a 54.5% (95% CI 48.8%-59.5%) in CAD risk.^7^ Such reduction is threefold higher than that achieved with the use of statins in more advanced age.^7^

Cholesterol metabolism can be analysed by dosing serum non-cholesterol sterols, cholesterol synthesis and absorption markers. In 1 to 10-year-old children, absorption prevails over synthesis.^8^ This finding shows the importance of dietary as a cholesterol-reduction tool among this age group.

In this issue of the *Arquivos Brasileiros de Cardiologia*, Gomes, et al.^9^ assessed the prevalence of isolated and combined dyslipidemias in 62,530 children and adolescents, aged between 1 day and 19 years, attended at the Basic Health Units network in Campinas/SP. They found biochemically classified changes in 67% of the lipid profiles analysed. The prevalence of increased total cholesterol, triglycerides, LDL and HDL-C levels were, respectively, 33%, 40%, 29% and 13%. The presence of low HDL-C was observed in 39% of the cases.^9^ Although the number of individuals analysed is a strength of this study, the exclusive analysis of the lipid profiles prevents any other conclusions besides the frequency of abnormalities in this population.

The risk factors present in childhood and adolescence will probably remain until adulthood. This period of life represents a window of opportunity to initiate effective measures aiming at the prevention of atherosclerosis and clinical outcomes in adulthood. Therefore, it is necessary to trace and treat abnormalities in the lipid profile of children and adolescents, particularly of those with other risk factors.

Thus, the *Updated Cardiovascular Prevention Guideline of the Brazilian Society of Cardiology - 2019* recommends the universal lipid screening between ages 9-11 and for children aged 2 years or older when other risk factors are present. The adoption of healthy eating habits, the practice of regular physical activity and weight control are the pillars of the treatment of dyslipidemia in this age group. The use of medication, predominantly statins, should be restricted to more severe cases (such as genetic dyslipidemias) and after unsuccessful non-pharmacological treatment.^10^
